# Sex-specific signatures of fetal programming for gestational diabetes mellitus in umbilical cord: A cohort study from Tanzania

**DOI:** 10.1186/s12916-026-05158-3

**Published:** 2026-08-21

**Authors:** Gad Hatem, Jonas Andersson, Line Hjort, Daniel T. R. Minja, Francesca Catellani, Sofie Lykke Møller, Olof Asplund, Dirk Lund Christensen, Birgitte Bruun Nielsen, Thor G. Theander, Isabella Artner, Nael Shaat, John P. A. Lusingu, Allan Vaag, Ib Christian Bygbjerg, Christentze Schmiegelow, Louise Groth Grunnet, Rashmi B. Prasad

**Affiliations:** 1 Lund University Diabetes Centre, Malmö, Sweden; 2 Department of Obstetrics, Center for Pregnant Women with Diabetes, Copenhagen University Hospital, (Rigshospitalet), Copenhagen, Denmark; 3 Novo Nordisk Foundation Center for Basic Metabolic Research, Metabolic Epigenetics Group, Faculty of Health and Medical Sciences, University of Copenhagen, Copenhagen, Denmark; 4 National Institute for Medical Research, Tanga Center, Tanga, Tanzania; 5 Global Health Section, Department of Public Health, University of Copenhagen, Copenhagen, Denmark; 6 Copenhagen University Hospital, Copenhagen, Denmark; 7 Centre for Translational Medicine and Parasitology, Department of Immunology and Microbiology, University of Copenhagen, Copenhagen, Denmark; 8 Copenhagen University Hospital, Steno Diabetes Center Copenhagen, Herlev, Denmark; 9 Department of Endocrinology, Skåne University Hospital, Malmö, Sweden; 10 Department of Gynaecology and Obstetrics, Copenhagen University Hospital– North Zealand, Hilleroed, Denmark; 11 Department of Clinical Sciences, Diabetes and Endocrinology, CRC, Lund University, Malmö, S-205 02 Sweden

**Keywords:** GDM, Fetal programming, Developmental programming, Tanzania, Epigenetic programming, Beta-cell development, Beta-cell function, Type 2 diabetes

## Abstract

**Background:**

Gestational diabetes mellitus (GDM) is defined as glucose intolerance first recognized during pregnancy. Offspring of GDM mothers have an increased risk of developing type 2 diabetes (T2D), obesity, and other metabolic disorders later in life.

**Methods:**

We analyzed metabolic, genetic, epigenetic, and transcriptional profiles in umbilical cord blood (UCB) from male and female offspring of GDM mothers versus normoglycemic controls in Tanzania. We constructed polygenic and methylation risk scores for T2D and GDM to assess whether diabetes-related susceptibility is detectable at birth. We identified differentially expressed genes (DEGs) and corresponding epigenome-wide alterations in GDM-exposed UCB. We then integrated genetic (PGS), epigenetic (MRS), and transcriptomic (DEG) data to identify convergent molecular signatures of GDM exposure. We examined their associations with neonatal anthropometry and cord insulin levels to infer potential effects on early growth. We further assessed DEG relevance by examining expression in human fetal and adult islets and by reviewing mouse-knockout data to explore possible roles in β-cell development or function.

**Results:**

Offspring of GDM mothers showed sex-specific differences in neonatal anthropometry, particularly among females, who exhibited greater adiposity and growth measures. Female offspring also displayed higher PGS for T2D, especially β-cell dysfunction, and a MRS for GDM/T2D associated with elevated cord insulin. We identified 36 DEGs in GDM-exposed UCB overall, with a striking sex-specific pattern (273 DEGs in females vs. 4 in males). Several DEGs and associated DNA methylation (DNAm) sites correlated with neonatal anthropometry, particularly in females, indicating putative fetal programming signatures. These signals showed convergence across genetic risk, DNA methylation, and gene expression layers. The expression of *SESN3*, *HMBS*, *NUTM2D*, and *MAN2A2* associated with insulin levels. *MAN2A2* expression also associated with birth weight and length in female offspring. The *SLC4A1* gene showed fetal pancreas-specific expression and co-expression with key pancreatic developmental genes, suggesting a role in pancreatic development.

**Conclusions:**

We identified integrated multi-omic signatures of GDM exposure in offspring at birth, with markedly stronger effects in females. These genes and pathways may contribute to fetal metabolic programming in this understudied Tanzanian population, offering new insights into biological mechanisms that may influence early development.

**Trial registration:**

ClinicalTrials.gov NCT02191683 Registered 2 July 2014.

**Graphical abstract:**

**Figure:**
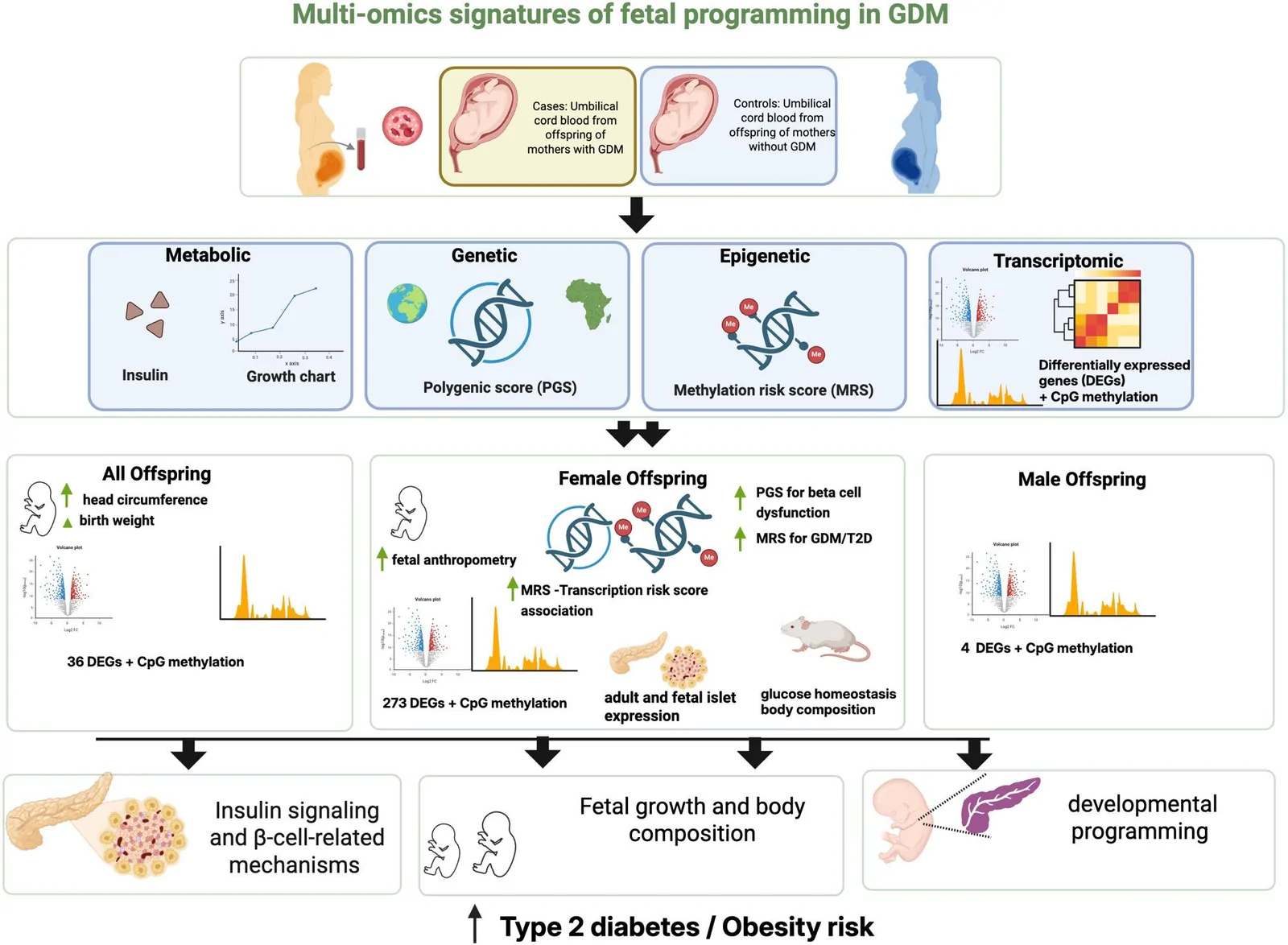

**Supplementary Information:**

The online version contains supplementary material available at 10.1186/s12916-026-05158-3.

## Background

Gestational diabetes mellitus (GDM) is hyperglycemia that is detected during pregnancy. Current worldwide prevalence estimates of GDM are 14% [1]. In Tanzania, reports ranged from 0% in a rural area in 1991(WHO 1985 criteria) [2] to 19.5% in urban areas in 2016 (WHO 2013) [3, 4] and our own study showed a 39% prevalence of GDM in Korogwe, rural northeastern Tanzania [5]. Glucose intolerance in pregnancy is a result of peripheral insulin resistance and insufficient β-cell expansion resulting in impaired maternal insulin secretion [6–10]. Previous studies have shown that maternal glucose crosses the placental interface, but insulin does not [11] and in GDM, this can result in fetal hyperglycemia and hyperinsulinemia [11]. This altered *in utero* environment has been shown to modify developmental programming in the fetus resulting in short as well as long term adverse consequences such as increased risk of type 2 diabetes (T2D) and obesity in the offspring [11, 12]. Moreover, evidence suggests sex-specific effects in fetal programming, with female offspring appearing more vulnerable to the metabolic consequences of maternal diabetes [13]. However, the molecular mechanisms underlying these observations remain hitherto unexplored.

Given the shared genetic component between GDM and T2D, the genetic predisposition reflected in the polygenic burden of metabolic traits may also differ in offspring of GDM mothers, potentially interacting with the intrauterine environment. Furthermore, there is evidence suggesting that epigenetic alterations play a role in developmental programming; however, most findings are based on association studies that highlight biomarker potential, while the underlying mechanisms remain unclear [14–20]. This focus stems from the recognition that early life represents a window of epigenomic plasticity that could especially modulate development. In addition to these epigenetic influences, changes in gene expression due to maternal hyperglycemia could in turn have consequences on fetal growth and development [15]. Although these blood-based epigenetic changes may not fully reflect alterations in the primary tissues, they hold significant potential as biomarkers and may provide valuable insights into the biological mechanisms operating in affected organs. To address these questions, we performed a metabolic, genetic, epigenetic and transcriptomic study of umbilical cord blood (UCB) from offspring of mothers with GDM and compared them to those from non-GDM controls to identify multi-omic alterations and molecular signatures associated with maternal hyperglycemia. We then examined whether these genes were linked to offspring growth by assessing associations with neonatal anthropometry. Finally, we investigated their expression in human embryonic pancreas and adult islets [21] as well as in knock-out mouse models from the International Mouse Phenotyping Consortium (IMPC) [22] to assess their association with development and function.

## Methods

### Study Participants

The FOETALforNCD cohort has been described in detail previously [23]. Briefly, the study was conducted in a rural region of North-eastern Tanzania, in Korogwe District and adjacent villages in Handeni District, Tanga Region from July 2014 to December 2016. In addition to the 100 UCB samples from newborns described previously [5, 20, 24], 44 newborn UCB samples from mothers with malaria were included in the present study (Additional file 1: Table S1, Additional file 2: Figure S1). Inclusion criteria for the main cohort in the presented analyses were: availability of both UCB and UCB collected in PAXgene tubes (for RNAseq), BW measured within 24 h of delivery, gestational age of > 22 weeks at delivery (≥ 155 days), HIV negative (unknown HIV status excluded), a live birth, not having any hypertensive disorders, and only singleton births (i.e., no twins or triplets). The UCB samples were collected in EDTA, serum and PAXgene tubes, primarily before the placenta was born. The collection tubes were placed in cold directly after collection and processed within 2 h, stored at − 20 °C for 24–72 h, and subsequently stored at − 80 °C [23]. GDM was diagnosed according to the WHO 2013 guidelines as follows: fasting venous plasma glucose 5.1–6.9 mmol/L, or venous plasma 1-h glucose value ≥ 10 mmol/L or 2-h glucose value 8.5–11.0 mmol/L. Over 94% of women were diagnosed with GDM based on a fasting blood sample [5]. The study was approved by regional ethical committees, and all participants gave written informed consent.

### RNA Isolation and sequencing

RNA was extracted from UCB stored in PAXgene tubes using the PAXgene Blood RNA Kit (Qiagen, catalog# 762174, Sollentuna). RNA quantity was assessed using the Agilent 4200 Tape station system and quantified using the Qubit assays (Thermofisher Scientific). DNA was extracted using the QIAamp DNA Blood Midi Kit (Qiagen, catalog # 51185, Sollentuna). DNA was quantified using picogreen assay (Promega) as described previously [20]. One µg of RNA (RNA integrity number RIN ≥ 7) was used for library preparation using the TruSeq Stranded Total RNA Library Prep Globin (Illumina). RNA sequencing was performed using the 75-bp paired-end protocol on a Nextseq 500 platform (Illumina) as previously described [20].

### RNA sequencing, preprocessing and analysis

Paired-end 101-bp length output reads were aligned to the human reference genome (hg38) using STAR (2.4.1a) [21]. The featureCounts script implemented as part of the Subreads software package (https://subread.sourceforge.net/) was used by counting uniquely mapped reads in each exon and gene [25]. Differential expression analysis on genes with quantifiable expression in cord blood from (a) all offspring, (b) male offspring and (c) female offspring. We adjusted for maternal age, offspring sex, gestational age at delivery, mid-upper arm circumference (MUAC), gravidity, batch, early pregnancy hemoglobin (Hb) levels, malaria exposure, and methylation data-derived blood cell composition (using the Houseman method [26–28]). False discovery rate (FDR)-adjusted p-values were used to define statistical significance in these primary differential expression analyses.

### DNA isolation and quantification, genome-wide genotyping and methylation

DNA was extracted from UCB buffy coats using the QIAamp DNA Blood Midi Kit (Qiagen, catalog # 51185, Sollentuna) and was quantified using picogreen assay (Promega). The Illumina Infinium OmniExpressExome-8 v1.4 chip was used for genotyping the DNA samples using Illumina iScan at the Department of Clinical Sciences, Clinical Research Centre, Lund University, Malmö, Sweden. Standard quality control was applied as described previously [20].

#### Genome-wide genotyping

Variants with a call rate < 99%, extreme heterozygosity (>|mean ± 3SD|), monomorphism, or low minor allele frequency (MAF < 1%) were excluded prior to imputation. Hardy–Weinberg equilibrium cut-offs of *p* < 5.7 × 10⁻⁸ for common and *p* < 10⁻⁴ for rare variants were applied. Quality control was conducted using **PLINK v1.9** [25]. After QC, 281,589 variants remained for imputation. Genotypes were aligned to the human genome reference (NCBI build 37) and imputed to the **1000 Genomes Phase 3** European reference panel (http://www.well.ox.ac.uk/~wrayner/tools/). Post-imputation, SNPs with MAF < 0.01 or imputation quality < 0.4 were excluded. **eQTL** analyses were performed in **MATRIX-QTL** following standard data transformations.

#### Methylation

Genome-wide DNA methylation was measured using the EPIC Bead Chip (WG-317-1001) with Infinium assay using the standard Infinium HD Assay Methylation Protocol Guide (part number 15019519, Illumina). Samples were randomly allocated across arrays. The EPIC BeadChip includes >850,000 probes, providing 99% coverage of RefSeq genes and accommodating 12 sam-ples per chip. Raw methylation intensities were extracted using the GenomeStudio Methylation Module (GenomeStudio Genome Browser, NCBI build 38), which calculates methylation β-values as:

$\:\beta\:=\frac{M}{(U+M+100)}$where *M* and *U* represent methylated and unmethylated signal intensities, respectively.

All samples passed GenomeStudio quality control metrics, including staining, hybridization, extension, and specificity controls, and exhibited high bisulfite conversion efficiency (signal intensity > 4000) [29]. Probes were excluded if they met any of the following criteria: detection *P* > 0.01, fewer than three beads in ≥ 5% of samples, non-CpG or SNP-related probes, multi-hit probes, or allosomal CpGs. After filtering, 749,969 probes remained.

Background correction and Beta MIxture Quantile normalization (BMIQ) were applied using **ChAMP** [30] to adjust for differences between type I and type II probes. Batch effects were assessed using singular value decomposition (SVD) and corrected using **ComBat** [31]. Because buffy coat DNA reflects a mixture of blood cell types, **RefbaseEWAS** was employed to correct for cellular heterogeneity based on reference methylation profiles from purified leukocyte subtypes [27, 28].

For differential methylation analysis, to stabilize variance in highly methylated or unmethylated sites, β-values were transformed to M-values using the **lumi** package [32], calculated as:

$\:M={\mathrm{l}\mathrm{o}\mathrm{g}}_{2}\left(\frac{\beta\:}{1-\beta\:}\right)$Covariates included maternal age, offspring sex, gestational age at delivery, mid-upper arm circumference (MUAC), gravidity, batch, early pregnancy hemoglobin (Hb) levels, malaria exposure, and methylation data-derived blood cell composition (using the Houseman method).

#### Statistics

The data were analyzed with Pearson correlation unless stated otherwise. Data are presented as the mean ± SD, unless stated otherwise. A P-value below 0.05 was considered statistically significant. Nominal associations were defined as those with unadjusted p-values < 0.05 and are reported for exploratory analyses, including associations with offspring anthropometric traits.

### Polygenic, methylation and transcription risk scores

Polygenic scores (PGS) were constructed based on single nucleotide polymorphisms (SNPs) associated with T2D in the largest multi-ancestry T2D GWAS till date [33]. SNP data were extracted chromosome-wise and subsequently merged to construct polygenic risk scores (PGS).

The 1289 SNPs, effect / risk alleles and beta-values were obtained and PGS for T2D as well as the eight clusters defined in Suzuki et al. [33] were constructed. In a second analysis, a subset of SNPs shown to associate with African T2D (predominantly of West African ancestry) as well as those assigned to the clusters were grouped to construct PGSs.

Each PGS was constructed summing the number of risk alleles weighted by their reported effect sizes. Only SNPs with available genotype data and consistent allele alignment were included. PGSs and continuous variables were rank transformed to obtain normal distribution. To evaluate the association between each PGS and GDM-exposure, firth logistic regression was used Standard linear regression was used for assessing the association between PGS and continuous traits such as birth weight, cord c-peptide and insulin values. Gestational age and offspring sex (for analysis including all offspring) were used as covariates.

To construct the weighted DNA methylation risk scores (MRS), we used summary data for the CpGs sites discovered in an EWAS of GDM in two cohorts of European and multi-ancestry background (*n* = 512) [34]. These CpG sites were also previously associated with T2D in large scale EWAS [35]. The regression coefficients for the EWAS of GDM was based in β-values. We calculated MRS for each of the discovered CpG sites available in our study. MRS was constructed by multiplying the regression coefficient from the GDM-EWAS with individual β-values. Thereafter we summarized the individual score for each of the CpGs sites to obtain the MRS. For association with GDM-exposed and continuous variables, logistic and linear regression was applied respectively with gestational age, offspring sex (for all offspring) and methylation derived cell composition.

A transcriptional risk score (TRS) was constructed as a weighted sum of gene expression levels for each of the analyses (all offspring, male offspring and female offspring), using case-control log fold-change estimates as weights for each gene. The resulting score was calculated for each individual by summing weighted expression values across all genes included in the signature. TRSs were rank transformed and associations between the TRS - PGS and TRS - MRS were assessed using multivariable linear regression models, adjusting for age, sex, and gestational age.

### Lookups

Gene expression look-up in human pancreatic islets was performed using RNA sequencing data derived from pancreatic islets of 191 donors from a previously published study [21]. Lookup in bulk and single-cell RNAseq of embryonic pancreas was performed and analyzed as previously described [36, 37] and in the Baron et al. dataset [38]. Comparison of expression in adult pancreas was performed using data from the GTEX database [39].

## Results

### Pronounced sex-differences in offspring anthropometry of GDM mothers

A total of 132 umbilical cord blood (UCB) samples were analyzed, including 48 from pregnancies complicated by gestational diabetes mellitus (GDM) and 84 from control pregnancies. Among the GDM group, 31 offspring were male and 17 were female. Mothers with GDM were older and exhibited higher fasting, 1‑hour, and 2‑hour glucose levels compared with non‑GDM mothers. Offspring born to GDM mothers had a larger head circumference and nominally higher birth weight than those born to non‑GDM mothers.

Sex‑specific differences in offspring anthropometry were more pronounced when comparing GDM and non‑GDM pregnancies. Female offspring of GDM mothers exhibited higher birth weight, head circumference, mid‑upper arm circumference, skinfold thickness, and chest circumference at delivery compared with female offspring of non‑GDM mothers. In contrast, no significant anthropometric differences were observed among male offspring (Additional file 1: Table S1).

### Genetic clusters reveal beta-cell-specific and sex-specific predisposition to impaired insulin secretion and T2D risk in GDM-exposed offspring

**Fig. 1:**
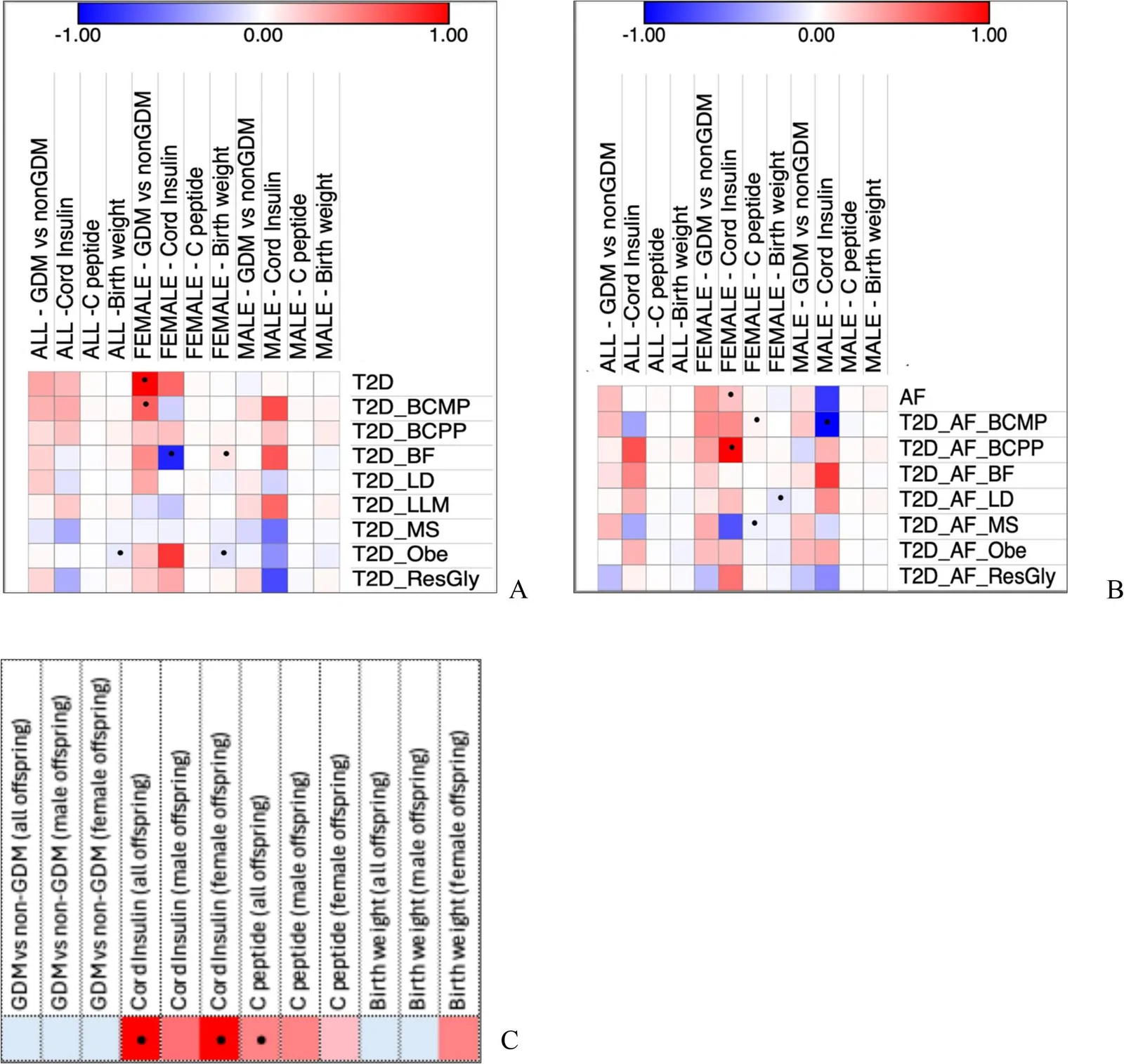
Heat map showing association of polygenic scores and methylation risk scores in GDM-exposed cord blood compared to controls. **A** Polygenic risk scores constructed from 1289 SNPs associated with T2D risk from Suzuki et al [33], as well as the 8 genetic clusters associated with cardiometabolic phenotypes. **B** Polygenic scores constructed from a subset of T2D risk SNPs associated with African T2D as well as the 8 genetic clusters constructed from African T2D risk loci. **C** Methylation risk score constructed from CpG sites whose methylation levels associated with T2D and GDM [34].

We assessed whether offspring born to mothers with GDM carry a higher polygenic burden for T2D. To this end, we constructed polygenic scores (PGS) using 1,289 SNPs associated with T2D risk identified in the largest multi-ancestry genome-wide association study (GWAS) to date [33]. The T2D polygenic score (T2D-PGS) was higher in offspring of GDM mothers, with this difference being evident in female but not male offspring. In addition, a PGS derived from African ancestry–specific T2D risk loci (a subset of the 1,289 SNPs) [33] was associated with cord insulin levels in female offspring (Additional file 1: Table S2; Fig. 1).

Suzuki et al. previously identified eight genetic clusters composed of non-overlapping SNPs associated with distinct cardiometabolic phenotypes [33]. To gain mechanistic insight, we next evaluated associations between cluster-specific PGS and GDM exposure status. Among female offspring, PGS reflecting beta-cell dysfunction; specifically, the beta-cell minus proinsulin and plus proinsulin cluster (T2D_BCMP_BCPP), as well as the beta-cell minus proinsulin cluster (T2D_BCMP) were significantly higher in GDM-exposed individuals compared with controls.

Consistent with these findings, analyses using African ancestry-specific T2D cluster PGS showed that beta-cell dysfunction clusters were associated with cord C-peptide levels in female offspring. Specifically, the AF-T2D_BCMP score was associated with lower cord insulin levels in male offspring, whereas the AF-T2D_BCPP score was associated with higher cord insulin levels in female offspring. Furthermore, the obesity cluster PGS was associated with birth weight in all offspring, while the lipodystrophy cluster PGS was associated with birth weight only among female offspring (Additional file 1: Table S2; Fig. 1).

### Methylation risk score for GDM and T2D associates with fetal insulin secretion

We next constructed a methylation risk score (MRS) based on 6 CpG sites previously associated with GDM (and T2D) in a multi-ancestry study [34, 35]. The MRS was associated with cord insulin in all offspring. In the sex-stratified analysis, the MRS was associated with cord insulin in female offspring but not male offspring. The MRS was also associated with C-peptide levels in all offspring (Additional file 1: Table S3, Fig. 1).

### Sex-specific differences in gene expression accompanied by DNA methylation in umbilical cord blood from offspring of mothers with GDM compared to controls

**Fig. 2:**
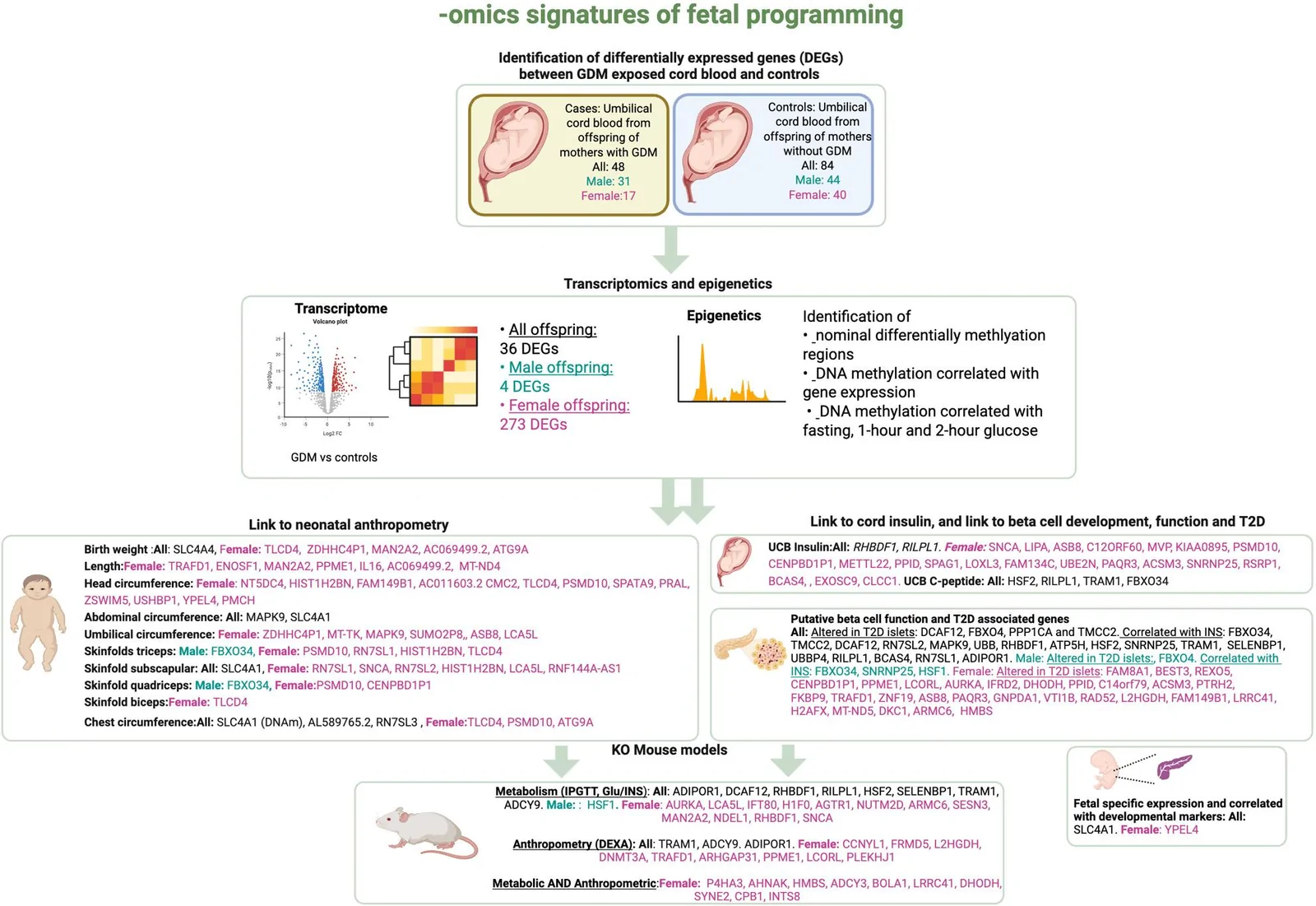
DEG-based integrative analyses in GDM-exposed offspring. Overview of study design and downstream analyses based on differentially expressed genes (DEGs) identified in umbilical cord blood from offspring exposed to gestational diabetes (GDM) and controls. DEGs were identified in the full cohort and in sex-stratified analyses. Integrated multi-omics analyses include transcriptomics and DNA methylation, highlighting nominally differentially methylated regions and correlations with gene expression and glycaemic traits (fasting, 1-hour, and 2-hour glucose).Downstream analyses link DEGs to neonatal anthropometric traits (including birth weight, length, circumferences, and skinfold measures) and cord blood insulin-related measures (insulin, C-peptide), as well as enrichment for pathways related to beta cell function and type 2 diabetes. Functional follow-up includes evidence from knockout (KO) mouse models for metabolic and anthropometric phenotypes, and assessment of fetal-specific expression and developmental marker associations. Gene names are shown, with sex-specific results indicated. Arrows depict the analytical workflow from DEG identification to downstream phenotypic and functional integration. *Created in BioRender. Prasad, RB. (2026)*

We performed a transcriptome-wide analysis on 48 UCB samples from mothers with GDM and 84 controls to identify potential fetal programming signatures of GDM (Fig. 1, Table S1). This analysis yielded 36 differentially expressed genes (DEGs) (Additional file 1: Table S4, Fig. 2). A similar analysis on 17 GDM-exposed vs. 40 controls **female** offspring revealed 273 DEGs whereas the corresponding analysis on 31 vs. 44 **male** offspring showed 4 DEGs (Table S1, S4, Fig. 2). Pathway analysis of the 273 DEGs in female offspring using Reactome [40] showed an overrepresentation of genes involved in RNA metabolism, gene expression and DNA repair pathways (Additional file 1: Table S5, Additional file 2: Figure S2).

CpG sites in several DEGs showed differential methylation (*p* < 0.05) between GDM exposed and non-GDM exposed UCB (Additional file 1: Table S6). Together, these findings indicate that DNA methylation changes at CpG sites within DEGs are functionally linked to gene expression, and that the MRS captures broader epigenetic variation that converges on these gene-specific methylation-expression relationships.

Across all offspring, DNA methylation at 29 CpG sites within six genes was significantly correlated with expression of the corresponding genes. Notably, methylation at CpG sites within *FBXO34*, *SLC4A1*, and *TMCC2* showed expression correlations at a greater number of sites than expected by chance alone (> 5%; Additional file 1: Table S7, Fig. 2).

In female offspring, DNA methylation at 95 CpG sites was significantly associated with expression of 31 corresponding genes (Additional file 1: Table S8). Methylation at several of these CpG sites was also associated with maternal fasting and 2‑h glucose concentrations, both in the overall cohort and in female offspring specifically (Additional file 1: Table S9-S12).

### Integration of transcriptional signatures with polygenic and methylation risk scores reveals sex‑specific associations

We next assessed whether polygenic (PGS) and methylation (MRS) risk scores were associated with differentially expressed genes (DEGs). To this end, we constructed a transcriptional risk score (TRS) for all offspring and in sex‑stratified analyses (male and female), calculated as a weighted composite of DEGs. Among the PGSs, only the African ancestry-specific T2D cluster PGS for body fat showed a nominal association with TRS in female offspring (*p* < 0.05) (Additional file 1: Table S13).

We then evaluated associations between TRS and MRS. The MRS was significantly associated with the TRS derived from the 273 DEGs in female offspring. In contrast, no significant associations were observed for TRS constructed from all offspring (36 DEGs) or from male offspring (4 DEGs).

At the individual gene level, the MRS was significantly associated with 7 of 36 DEGs (19.4%) in all offspring (*p* < 0.05). In female offspring, 12 DEGs were significantly associated with MRS, and a larger proportion (166 of 273 DEGs; 60.8%) showed nominal associations (*p* < 0.05) (Additional file 1: Table S13). The proportion of DEGs associated with MRS was significantly higher in female offspring compared with all offspring (60.8% vs. 19.4%; OR = 6.43, 95% CI 2.72–15.19, Fisher’s exact test *p* = 3.3 × 10⁻⁶), indicating markedly stronger enrichment of MRS-transcriptional associations in females. No associations were observed in male offspring. These findings provide a direct link between the externally derived methylation risk score (MRS) and gene-specific DNA methylation and expression changes in our dataset, as the TRS is derived from DEGs that are themselves associated with CpG methylation levels.

### Association with offspring anthropometry

**Fig. 3:**
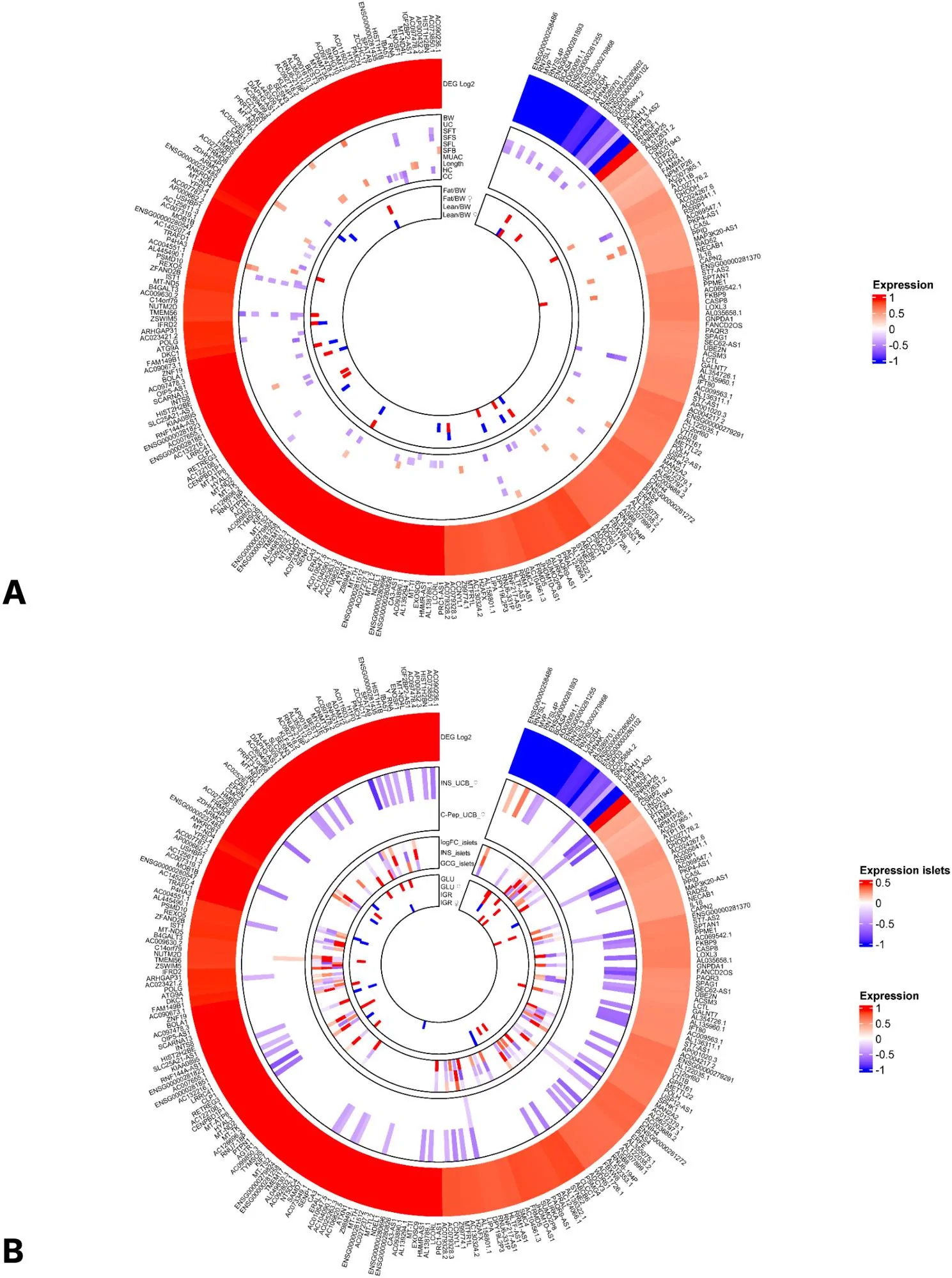
Overview findings in female offspring. **A** circular plot showing DEGs and their associations with neonatal anthropometry and corresponding rodent KO data for body composition. DEG_LogFC = logFC for differentially expressed genes (DEGs) in umbilical cord blood (UCBs) of female off-spring from GDM mothers compared to controls. Association of DEGs with neonate: BW = birth weight, UC = abdominal circumference at umbilical level at delivery, SFT = skinfold triceps at de-livery, SFS = skinfold subscapular at delivery, SFL = skinfold quadriceps at delivery, SFB = skin-fold biceps at delivery, MUAC, = mid-upper arm circumference Length = delivery length of neo-nate, HC = head circumference, CC = chest circumference, Rodent KO readout for DEGS : Fat/BW = fat by body fat ratio, Lean/BW = lean by body fat ratio. **B** circular plot showing DEGs and their association with insulin and C-peptide and corresponding rodent KO data for metabolism. DEG Log2 = log2 fold change of differentially expressed genes (DEGs) in UCB from GDM moth-ers compared to controls. Association of DEGs with neonate: INS_UCB = insulin in UCB, C-pep_UCB = C-peptide in UCB. logFC_islets = log fold change of the DEGs in T2D donor islets comparted to controls from Asplund et al, INS_islets = correlation of DEGs with insulin expression in islets, GCG = correlation of DEGs with glucagon expression in islets, Rodent KO readout for DEGS : GLU = intraperitoneal glucose tolerance test area under glucose response curve (IPGTT_AUCG), Fasted blood glucose concentration (FG), or Glucose , IGR = Initial response to glucose challenge

In all offspring, the expression of six genes was nominally associated (*p* < 0.05) with measures of neonatal anthropometry whereas in sons, one gene (*FBXO34*) showed such an association. In female offspring, the expression 70 DEGS showed nominal associations with at least one neonatal anthropometric measure. Notably, *TLCD4* expression was associated with birth weight, head circumference, chest circumference, and the sum of triceps and biceps skinfolds. The expression of *PSMD10* was associated with five anthropometric measures, while *HIST1H2BN* expression was associated with three (Additional file 1: Table S14; Figs. 2 and 3).

Several of these genes were accompanied by CpG sites for which DNA methylation was also nominally associated with the corresponding anthropometric measures (*p* < 0.05; Additional file 1: Table S15). Among these, DNA methylation at cg15982372 and cg20830361 within *PRC1‑AS1* and *MAPK9*, respectively, was significantly associated with head circumference and umbilical circumference after correction for multiple testing (FDR < 0.05; Additional file 1: Table S15). DNA methylation at several of these CpG sites was additionally associated with gene expression (Additional file 1: Tables S9 and S14; Fig. 2).

### Association with UCB insulin and C-peptide levels

The expression of *RHBDF1* and AD000091.1 was nominally associated with UCB insulin levels (*p* < 0.05; Additional file 1: Table S16). DNA methylation at CpG sites within *RHBDF1*, *TRAM1*, *SNRNP25*, and *SLC4A1* was nominally associated with insulin levels, while methylation at CpG sites within *HSF2* and *FBXO34* was nominally associated with C‑peptide levels (Additional file 1: Table S17; Fig. 1).

In female offspring, expression of 66 DEGs was associated with insulin levels, of which five remained significant after correction for multiple testing (FDR < 0.05; Table S16). Among these genes, DNA methylation at 30 CpG sites across 18 genes was correlated with insulin levels, whereas methylation at three CpG sites within *SNCA* was correlated with C‑peptide levels (*p* < 0.05; Additional file 1: Table S17; Fig. 2).

### eQTL analysis and look up of DEGs in previous GWAS for signals of association

To further investigate the genetic regulation of the DEGs, we performed expression quantitative trait loci (eQTL) analyses in all offspring and identified three genes with genome-wide significant eQTLs for expression in umbilical cord blood (UCB; Additional file 1: Table S18). In addition, four loci within *CLEC2B* were nominally associated with diabetes risk, while three additional loci were associated with indices of insulin secretion (Additional file 1: Table S19).

Among the DEGs, loci within *DCAF12*, *RILPL1*, and *SELENBP1* showed significant associations with type 2 diabetes in previous genome-wide association studies (GWAS), whereas *ADIPOR1* demonstrated a nominal association. Furthermore, loci within *RHBDF1*, *BCAS4*, *ADCY9*, and *SNRNP25* were significantly associated with glycemic traits, including random glucose and HbA1c. Multiple additional DEGs also exhibited strong associations with anthropometric traits [41].

### Expression of DEGs in adult pancreatic islets

To further link DEG expression with insulin secretion and islet function, we examined RNA‑sequencing data from pancreatic islets of 191 donors from a previous study [21]. Of the DEGs identified in all offspring, the expression of 19 was detectable in islets (Additional file 1: Table S20).

*FBXO34* and *DCAF12* were downregulated in islets from diabetic compared with non‑diabetic donors and showed negative correlations with insulin (*INS*) expression (Table S21). In contrast, *PPP1CA* and *TMCC2* were upregulated in diabetic donor islets; *PPP1CA* expression correlated negatively with glucagon (GCG), whereas *TMCC2* expression correlated positively with *INS* (Additional file 1: Table S20).

Overall, expression of 17 DEGs correlated significantly with *INS* expression and 12 with *GCG* expression. Eleven genes downregulated in UCB from offspring of GDM mothers showed negative correlations with *INS* and positive correlations with *GCG* expression, including *DCAF12*, *MAPK9*, *SELENBP1*, *ADIPOR1*, *FBXO34*, and *TRAM1* (Additional file 1: Table S20; Fig. 2). Conversely, *RHBDF1* and *RILPL1*, which were upregulated in UCB from offspring of GDM mothers, showed positive correlations with *INS* and negative correlations with *GCG*.

In male offspring, three of four DEGs were expressed in islets, including *FBXO34; SNRNP25* and *HSF1* expression correlated with *INS*, and *HSF1* also correlated with *GCG* (Additional file 1: Table S21). Among female offspring, 124 DEGs were expressed in islets, of which 32 were differentially expressed between T2D donors and controls in the same direction as observed in UCB. Expression of 103 DEGs correlated with *INS*, and 79 with *GCG* expression (Additional file 1: Table S21; Fig. 2).

### Developmental expression of DEGS in fetal and adult pancreas

To explore the potential developmental and functional relevance of the DEGs, we examined their expression in 16 fetal and 328 adult pancreatic samples from GTEx [39]. Genes uniquely or preferentially expressed in the fetal pancreas may reflect roles in pancreatic development, particularly β-cell differentiation and mass, which can influence later susceptibility to diabetes.

To identify fetal-specific expression, we defined detectable expression as ≥ 1 CPM in at least 75% of fetal samples and < 1 CPM in at least 75% of adult pancreatic samples. Using these criteria, S*SLC4A1* emerged as the only DEG with expression specific to the fetal pancreas.

As *SLC4A1* expression was not detectable in adult human pancreatic islets, we next examined RNA-sequencing data from sorted fetal and adult α- and β-cells (Blodgett et al. [4]). *SLC4A1* expression was significantly higher in fetal β-cells compared with adult β-cells (t = 4.51, *p* = 0.00044), whereas no significant differences were observed between fetal α- and β-cells or between fetal α- and adult α-cells.

We further evaluated *SLC4A1* expression single-cell RNA-sequencing data from embryonic pancreas at 8 weeks post-conception (Carnegie stage 22). Of 2,630 cells, 88 expressed *SLC4A1* (Fig. 3). Consistent with a potential developmental role, *SLC4A1* expression correlated significantly with key pancreatic developmental transcription factors *PDX1* (ρ = -0.29, *p* = 0.0067), *FOXA2* (ρ = -0.23, *p* = 0.034), and *NKX6-1* (ρ = -0.25, *p* = 0.017).

**Fig. 4:**
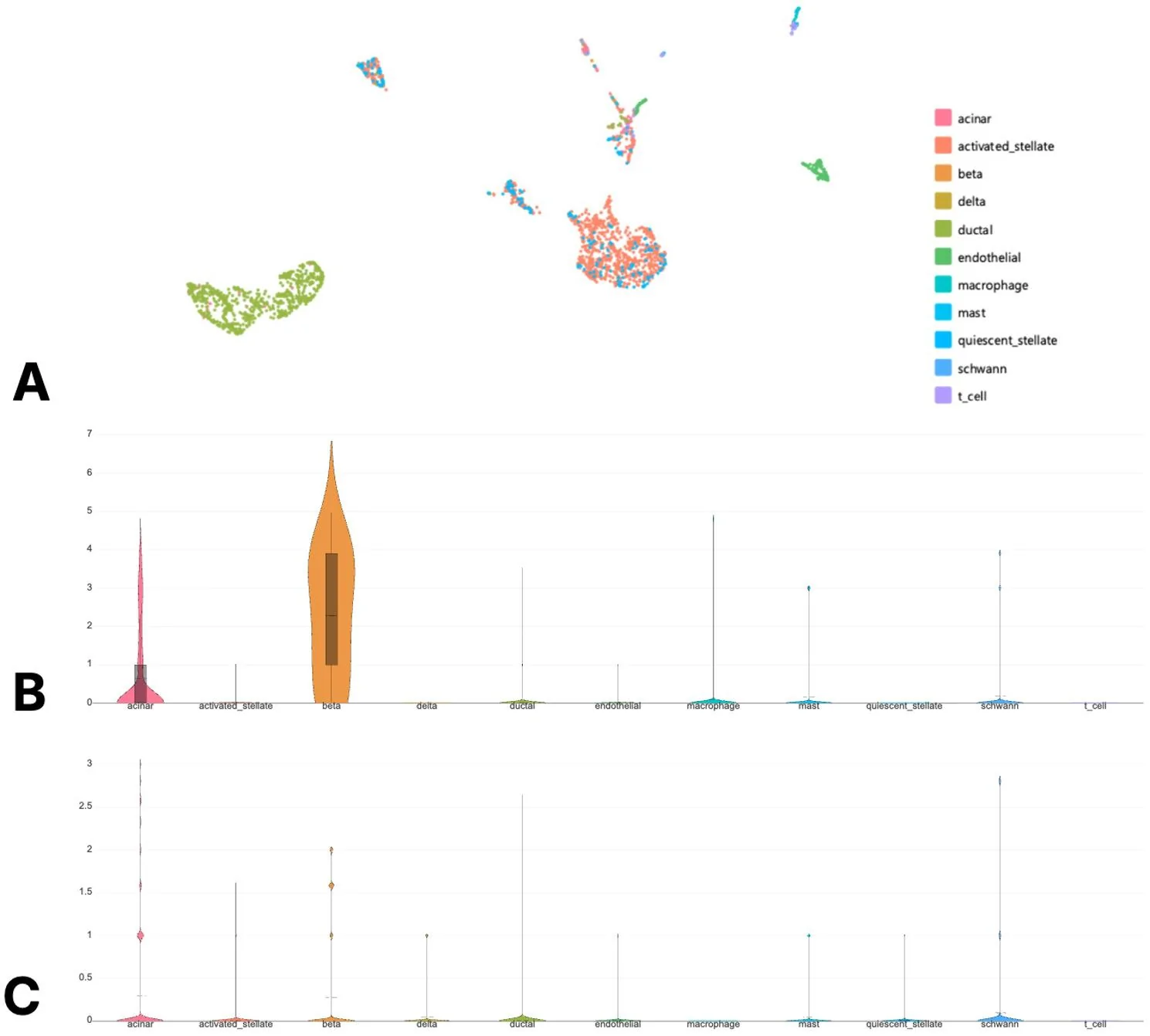
(**A**) Cell types expression in embryonic pancreas. cell-type clustering and annotation based on reference expression profiles, using established pancreatic cell-type signatures from Baron et al. Cells are grouped according to similarity in gene expression and assigned to canonical cell types (e.g., alpha, beta, delta, ductal, acinar, and other endocrine/exocrine populations). **B** Cell types were defined based on Baron et al [38]. Top violin plot shows expression of *SLC4A1* in embryonic alpha and beta-cells. **C** Violin plot at the bottom shows low expression of *YPEL4 *in all cell types

In female offspring, eight DEGs exhibited fetal pancreas-specific expression; among these, *YPEL4* showed no detectable expression in adult islets. Although *YPEL4* expression did not correlate with established developmental markers, it was detected in single-cell embryonic pancreas data across multiple cell types, including pancreatic progenitors and β-cells (Fig. 4), suggesting a potential role during early pancreatic development.

### DEGs influence metabolic phenotypes in vivo

To evaluate in vivo evidence for metabolic roles of the identified DEGs, we queried the International Mouse Phenotyping Consortium (IMPC) database [42](data accessed Nov 15, 2025). For DEGs identified in all offspring, knockout (KO) mouse models were available for 15 of 36 genes, with metabolic phenotype data for nine strains (Additional file 1: Table S22).

**Fig. 5:**
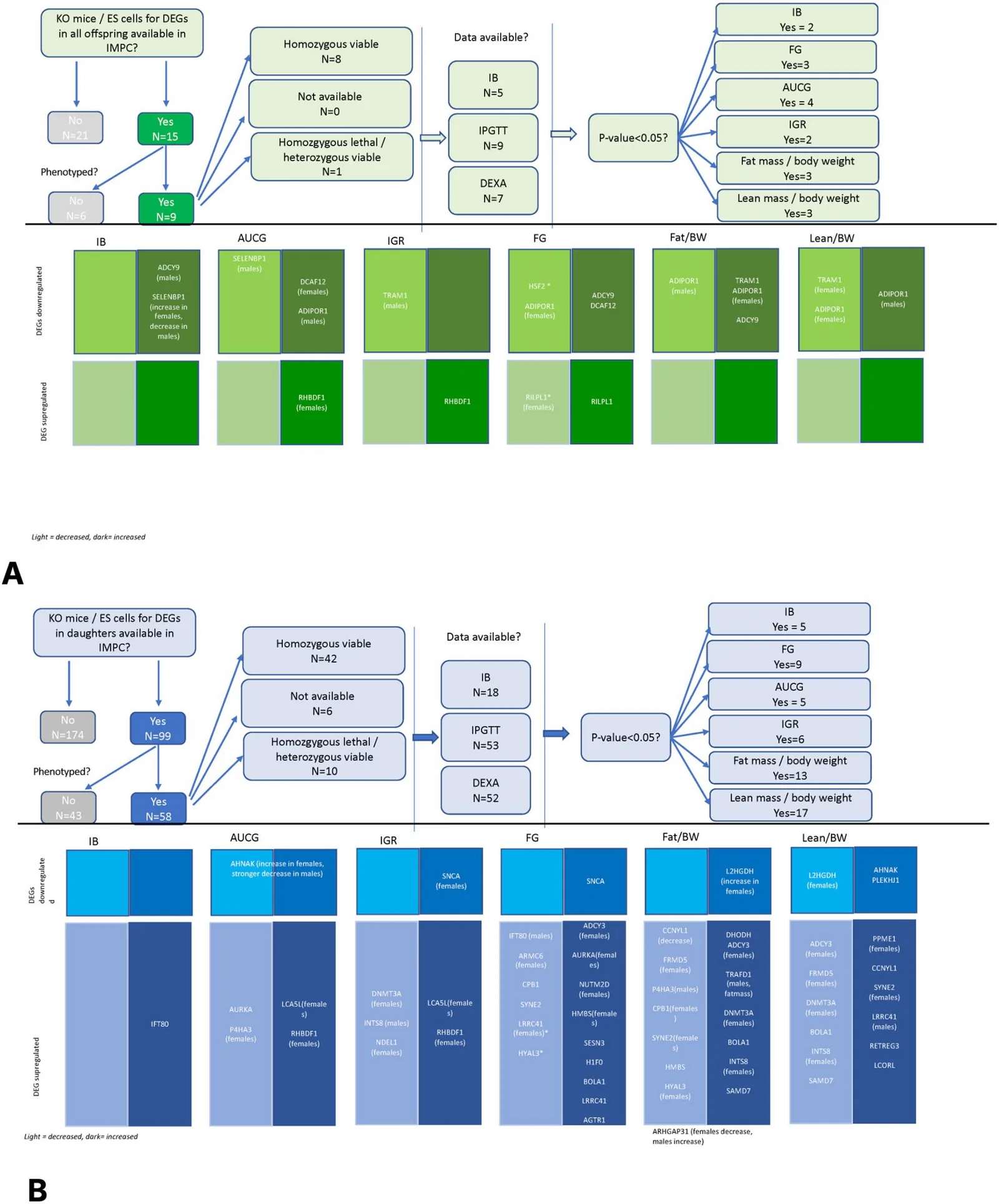
**A** Flowchart showing mice knock-out gene model data from the International Mouse Phenotyping Consortium (IMPC) for the differentially expressed genes (DEGs). Figure 4A. Summary of IMPC phenotype data for viable mice KO strains for genes showing differential expression in umbilical cord blood (UCB) from offspring of GDM mothers exhibit metabolic phenotypes. Figure 4B. Sum-mary of IMPC phenotype data for viable mice KO strains for genes showing differential expression in umbilical cord blood (UCB) from female offspring of GDM mothers exhibit metabolic pheno-types. IB: insulin blood levels, IPGTT: intraperitoneal glucose tolerance test, AUCG: Area under glucose response curve, IGR: Initial response to glucose challenge, FG: Fasted blood glucose con-centration, DEXA: Dual-energy X-ray absorptiometry, BW: Body weight. **B** Figure 4B. Summary of IMPC phenotype data for viable mice KO strains for genes showing differential expression in umbilical cord blood (UCB) from female offspring of GDM mothers ex-hibit metabolic phenotypes. IB: insulin blood levels, IPGTT: intraperitoneal glucose tolerance test, AUCG: Area under glucose response curve, IGR: Initial response to glucose challenge, FG: Fasted blood glucose concentration, DEXA: Dual-energy X-ray absorptiometry, BW: Body weight

Among these, insulin blood measurements were available for five KO strains, of which *ADCY9* and *SELENBP1* showed altered insulin levels (*p* < 0.05; Fig. 5A). Intraperitoneal glucose tolerance test (IPGTT) data were available for nine strains and revealed altered fasting glucose, glucose area under the curve, and/or overall glucose levels in *RILPL1*, *SELENBP1*, and *ADIPOR1* KO mice (*p* < 0.05; Table S22). In addition, the initial glucose response was altered in *RHBDF1* and *TRAM1* KO mice (*p* < 0.05). Dual‑energy X‑ray absorptiometry (DEXA) data were available for seven models, with altered total fat mass observed in *ADCY9*, *TRAM1*, and *ADIPOR1*, and altered lean mass in *TRAM1* and *ADIPOR1* (*p* < 0.05; Additional file 1: Table S22; Fig. 5A).

For male‑specific DEGs, a KO model was available for *HSF1*. *IPGTT* analyses showed reduced glucose levels and an increased initial glucose response in KO mice (Additional file 1: Table S22).

For DEGs identified in female offspring, KO models were available for 99 of 273 genes, with metabolic phenotypes reported for 58 strains (Fig. 4B). Insulin measurements were available for 18 KO strains, with altered levels observed in IFT80 (*p* < 0.05). IPGTT data from 53 KO strains showed altered fasting glucose in nine strains and altered glucose area under the curve in five strains (*p* < 0.05), including female‑specific effects for *SNCA*, *P4HA3*, *LCA5L*, *RHBDF1*, *ARMC6*, *LRRC41*, *ADCY3*, *AURKA*, and *NUTM2D* (Additional file 1: Table S22; Figs. 2 and 5B). Initial glucose response was altered for six genes, with female‑specific effects observed for *DNMT3A*, *NDEL1*, *LCA5L*, *SNCA*, and *RHBDF1*, and a male‑specific effect observed for *INTS8* (Additional file 1: Table S22).

DEXA body‑composition data were available for 52 KO models, of which 16 and nine exhibited altered fat mass and lean mass, respectively (*p* < 0.05). Female‑specific effects were observed for *FRMD5*, *L2HGDH*, *CPB1*, *SYNE2*, *HYAL3*, *ADCY3*, *DNMT3A*, *INTS8*, and *PPME1* (Additional file 1: Table S21; Figs. 2 and 5B).

Together, these findings from mouse KO models support a role for the identified DEGs in regulating glucose homeostasis and body composition in vivo. Collectively, these findings indicate that insulin signaling, β-cell-related pathways, and insulin-mediated growth represent the primary axes of convergence across genetic, epigenetic, transcriptomic, and phenotypic levels.

## Discussion

In this study, we uncovered fetal programming signatures related to metabolism, anthropometry, and T2D by integrating gene expression, DNA methylation, and genotyping data from umbilical cord blood (UCB) of Tanzanian offspring born to mothers with or without GDM. Maternal GDM was associated with greater birth size predominantly in female offspring, indicating marked sex dimorphism in fetal growth responses. Both polygenic and methylation risk scores influenced fetal insulin regulation, with beta-cell-related genetic risk and GDM-associated methylation patterns showing sex-specific effects. These findings were paralleled by transcriptional changes, with substantially more DEGs in females than males, and by corresponding DNA methylation alterations. Many of these molecular features were associated with neonatal anthropometry and enriched in fetal and pancreatic tissues where they were where they were altered in T2D donor islets and linked to insulin levels. Supporting their functional relevance, mouse knockout models demonstrated roles in metabolism and body composition, often reflecting the observed sex-specificity.

Both genetic and epigenetic variation appear to shape fetal insulin regulation in the context of maternal hyperglycemia. Offspring of GDM mothers exhibited higher polygenic scores for T2D, with enrichment in beta cell-related genetic clusters, particularly among females, suggesting a sex-specific genetic predisposition to impaired insulin secretion. Complementing this, a methylation risk score derived from CpG sites previously linked to GDM and T2D was associated with cord insulin and C-peptide levels, indicating that epigenetic marks reflecting maternal glycemic exposure also contribute to altered fetal insulin dynamics. Together, these findings highlight the combined influence of inherited and environmentally induced molecular risk factors on early-life metabolic programming, with notable sex-dependent effects.

GDM has been shown to have effects on both maternal and fetal health [43, 44] and previous studies have reported GDM‑associated changes in cord blood DNA methylation that can influence gene expression [17, 45]. In our cohort, DNA methylation at multiple CpG sites correlated with both gene expression and maternal glucose levels, supporting a role for intrauterine hyperglycaemia in transcriptional reprogramming. Although we did not replicate specific CpG sites reported previously, differences in cohort size, ancestry, and environmental context likely contribute to population‑specific methylation signatures.

Sex‑specific differences in fetal growth and metabolic responses to maternal hyperglycemia have been widely reported, with female offspring often showing greater adiposity and metabolic alterations later in life [46–54]. Although some studies have suggested that male offspring may be more susceptible to adverse intrauterine environments [55], vulnerability appears to be exposure‑ and outcome‑specific [56, 57]. Consistent with this, we observed that transcriptional and epigenetic responses to GDM were largely restricted to female offspring and were associated with differences in neonatal anthropometry. Although increased birth weight is a common outcome of GDM [58], significant differences in neonatal anthropometry in our study were observed primarily among females, extending beyond birth weight to measures of body composition. The expression of several DEGs and methylation at associated CpG sites correlated with these anthropometric traits in females but not in males, supporting a sex‑specific link between molecular variation and growth. These findings are consistent with sex‑dependent placental and metabolic adaptations [59, 60]. Female placentas exhibit greater transcriptional and epigenetic plasticity, which may enhance nutrient transfer and fetal insulin exposure, while differences in placental hormone signaling and maternal steroid profiles may further contribute to sex‑specific metabolic programming [51, 61–64]. Hormones produced by the placenta, such as estrogen and progesterone, can differentially influence glucose metabolism in male and female fetuses [65]. These hormones play a crucial role in regulating fetal development by influencing the development and function of various organs and systems, including the endocrine system, which includes the regulation of glucose metabolism. Previous studies have shown that exposure to high levels of estrogen during fetal development may affect glucose metabolism in female offspring and an increased risk of developing insulin resistance and glucose intolerance later in life [63]. Previous studies have also shown that a high fat diet in mothers can lead to epigenetic modifications in the genes of female offspring, potentially affecting their metabolic regulation and increasing their risk of developing diabetes later in life [64]. While this was not the case in ‘our’ mothers, whose diet was not high in fat, however, high levels of glucose *in utero* might potentially influence the programming of metabolic processes differently in males and females. Together, these differences suggest distinct adaptive strategies rather than uniform vulnerability and align with the pronounced female‑specific effects observed in our study.

The pronounced sex-specific differences observed in gene expression and epigenetic markers warrant careful consideration. While previous studies have reported sex-related variation in fetal growth and metabolic responses to maternal hyperglycemia [48–54], the magnitude of transcriptional differences in our cohort was unexpected. To minimize the possibility of chance findings, we applied stringent FDR correction and confirmed pathway-level enrichment for insulin signaling and β-cell development among female DEGs. These observations further support sex‑specific differences in placental function and hormonal milieu, which may influence fetal sensitivity to maternal glucose exposure [63–65]. Nevertheless, given the limited sample size and imbalance in sex distribution, replication in larger, independent cohorts is essential to confirm these findings and clarify whether genetic risk score differences contribute to the observed patterns. Our results should therefore be interpreted as hypothesis-generating, highlighting potential mechanisms that merit further investigation.

Another important consideration is whether the observed sex‑specific findings could be explained by differential transmission of polygenic risk to female offspring, potentially rendering downstream molecular associations coincidental. However, polygenic risk alleles are transmitted independently of offspring sex, and sex‑specific effects are more plausibly driven by differential penetrance within sex‑dependent fetal physiological contexts. Moreover, our findings extend beyond polygenic risk alone: transcriptional changes were accompanied by concordant DNA methylation patterns, associated with neonatal anthropometry, enriched in fetal and adult pancreatic tissues, altered in islets from individuals with type 2 diabetes, and supported by metabolic phenotypes in mouse knockout models. Such convergence across molecular layers, tissues, developmental stages, and species is unlikely to arise from stochastic inheritance alone and instead supports a biologically structured, sex‑dependent fetal response to maternal hyperglycemia.

Offspring of mothers with GDM were enriched for PGS related to for β‑cell dysfunction which associated with fetal insulin levels and aligned with transcriptional changes in genes linked to insulin secretion and metabolic pathways. They also exhibited higher insulin and C‑peptide levels in UCB, while not statistically significant, potentially reflected fetal β‑cell responses to increased maternal glucose exposure. Accordingly, expression of several DEGs was nominally associated with insulin and C‑peptide levels, with pronounced sex‑specific effects: associations were observed for three DEGs in all offspring and for 66 and eight DEGs with insulin and C‑peptide, respectively, in female offspring, whereas no such associations were observed in males. Many of these expression changes were accompanied by corresponding DNA methylation differences, suggesting potential epigenetic mediation. Several DEGs, including *SESN3*, *HMBS*, *NUTM2D*, and *MAN2A2*, showed concordant associations with insulin levels in UCB and in adult pancreatic islets, and some were differentially expressed in islets from individuals with T2D [21]. Functional support was further provided by knockout mouse models demonstrating altered glucose homeostasis, insulin responses, and body composition. Consistent with insulin’s role as a key fetal growth factor, genes such as *MAN2A2*, *LCA5L*, *SNCA*, and *PPME1* were also associated with neonatal anthropometry, including birth weight and length, particularly among female offspring. Together, these findings suggest that the identified DEGs represent fetal programming signatures linking altered insulin secretion to sex‑specific growth trajectories and increased susceptibility to cardiometabolic disease later in life [62].

We further hypothesized that genes influencing fetal development may contribute to fetal programming with long‑term metabolic consequences. Accordingly, DEGs expressed in the fetal but not adult pancreas may play roles in pancreatic development. We identified *SLC4A1* as being specifically expressed in the fetal pancreas but not in adult pancreas or islets. Its expression correlated with key pancreatic developmental transcription factors, including *PDX1*, *FOXA2*, and *NKX6‑1*, suggesting a potential role in pancreatic development. *SLC4A1* was also nominally associated with neonatal anthropometry. Although mutations in *SLC4A1* are classically linked to distal renal tubular acidosis [66] and hereditary spherocytosis [67], the gene has also been implicated in resistance to Plasmodium species [68]. While malaria exposure in our cohort may contribute to its differential expression, this does not readily explain its specific enrichment in fetal pancreas compared with adult tissue, supporting a potential developmental function.

Despite the large number of associations identified, these findings converge on a limited number of biologically coherent pathways. Most prominently, we observed consistent evidence implicating insulin signaling and β‑cell-related mechanisms across genetic, epigenetic, transcriptomic, and phenotypic levels. Polygenic scores enriched for β‑cell dysfunction were associated with cord insulin levels. In parallel, a methylation risk score derived from GDM‑ and T2D‑associated CpG sites was associated with fetal insulin secretion, and several DEGs showed concordant associations with insulin and C‑peptide, islet expression, and metabolic phenotypes in knockout mouse models. Notably, integration of transcriptional and epigenetic data further supported this convergence, as methylation risk scores were significantly associated with transcriptional risk scores derived from DEGs in female offspring, with a markedly higher proportion of DEGs showing methylation associations compared with the overall cohort. This enrichment was restricted to female offspring and not observed in males, reinforcing the sex‑dependent coupling between epigenetic variation and transcriptional regulation. Importantly, while the MRS is derived from CpG sites identified in independent cohorts, our data show convergence with gene-specific DNA methylation changes at DEGs, suggesting that the MRS reflects broader, environmentally induced epigenetic variation that manifests at functionally relevant loci in our dataset. These findings also reflect the combined influence of inherited and environmentally induced molecular variation, as both polygenic risk for β‑cell dysfunction and GDM‑associated methylation patterns influenced fetal insulin regulation, particularly in female offspring.

A second axis involved insulin‑mediated fetal growth, with female‑specific differences in anthropometry paralleled by transcriptional and epigenetic variation in genes associated with birth size and adiposity. These findings support a role for altered fetal insulin signaling in mediating growth responses to maternal hyperglycemia.

Finally, a subset of genes showed evidence of developmental programming, including fetal‑specific pancreatic expression and regulation by maternal glucose. These findings suggest that maternal GDM may perturb pancreatic developmental processes during critical windows of fetal plasticity. Together, these findings indicate that maternal hyperglycemia primarily programs fetal insulin secretion and growth through coordinated genetic, epigenetic, and transcriptional mechanisms, with stronger effects in female offspring.

This study represents a pilot investigation of GDM‑associated fetal programming in a rural Tanzanian population. Despite the multi‑layered consistency observed across molecular and phenotypic levels, several limitations should be considered. Limitations include modest sample size, sex imbalance, and the absence of long‑term follow‑up data in offspring. While neonatal anthropometry provides a well‑established proxy for future metabolic risk, longitudinal studies will be required to determine long‑term outcomes. Importantly, false discovery rate (FDR) correction was applied to define statistical significance in the primary analyses, including differential gene expression and methylation analyses, whereas associations with offspring anthropometry were based on nominal p‑values and should be interpreted as exploratory. Importantly, although these analyses are exploratory, the consistent convergence of findings across multiple independent data layers (gene expression, DNA methylation, genetic risk scores, and functional data) supports the biological relevance of these associations. All reported findings are associative, and causal mechanisms remain to be established. Future studies with larger cohorts, parental genotyping, and experimental validation will be essential to disentangle inherited and environmentally driven contributions to developmental programming.

## Conclusions

We identified candidate molecular signatures linking maternal GDM exposure to fetal metabolic and growth programming in a Tanzanian population. These results underscore the intrauterine period as a critical window for sex‑specific metabolic reprogramming and suggest potential targets for early‑life interventions aimed at reducing later cardiometabolic risk.

## Supplementary Information

Below is the link to the electronic supplementary material.

Supplementary Material 1: Additional file 1: Tables S1-S22

Supplementary Material 2: Additional file 2: Figure S1-S2

Supplementary Material 3: Additional file 3

## Data Availability

The UCB datasets generated for this study (DNA methylation data: accession number LUDC2026.05.1, mRNA data: accession number LUDC2026.05.2, and GWAS data: accession number LUDC2026.05.3) are deposited in the LUDC repository (https://www.ludc.lu.se/resources/repository). Data from the UCB are available upon request and acceptance from the FoetalforNCD Data Access Committee (contact author for application). The processed mRNA-seq expression matrix underlying the analyses presented in this study is provided as Additional File 3. Individual-level data from the terminated fetuses are not publicly available due to ethical and legal restrictions related to the Swedish Biobanks in Medical Care Act, the Personal Data Act, and the European Union’s General Data Protection Regulation and Data Protection Act. The dataset of human pancreatic islet samples from subjects with T2D or IGT and controls is publicly available at https://mae.crc.med.lu.se/IsletGeneView/ (Islet Gene View).

## Abbreviations

AUCG: Area Under the Curve for glucose
BMI: Body mass index
BMIQ: Beta mixture quantile normalization
CPM: Counts per million
DEG: Differentially expressed gene
DEXA: Dual-energy x-ray absorptiometry
DNAm: DNA methylation
eQTL: Expression quantitative trait loci
FDR: False discovery rate
FG: Fasting blood glucose concentration
GDM: Gestational diabetes mellitus
Hb: Hemoglobin
IMPC: International Mouse Phenotyping Consortium
IB: Insulin blood
IGR: Initial response to glucose
INS: Insulin
IPGTT: Intraperitoneal glucose tolerance test
lncRNA: Long non-coding RNA
MUAC: Mid-upper arm circumference
NK cells: Natural Killer cells
qCML: Quantile-adjusted conditional maximum likelihood
RCH clinic: Reproductive and child health clinic
SNP: Single-nucleotide polymorphism
SVD: Singular value decomposition
T2D: Type 2 diabetes mellitus
UCB: Umbilical cord-blood
