# Supplementary material for: Sex-specific signatures of fetal programming for gestational diabetes mellitus in umbilical cord: A cohort study from Tanzania
Source: BMC Med. 2026 Aug 21;24:457. doi: 10.1186/s12916-026-05158-3 (PMC13499328; doi:10.1186/s12916-026-05158-3)
Supplement: Supplementary file 1 [file 12916_2026_5158_MOESM2_ESM.pdf]

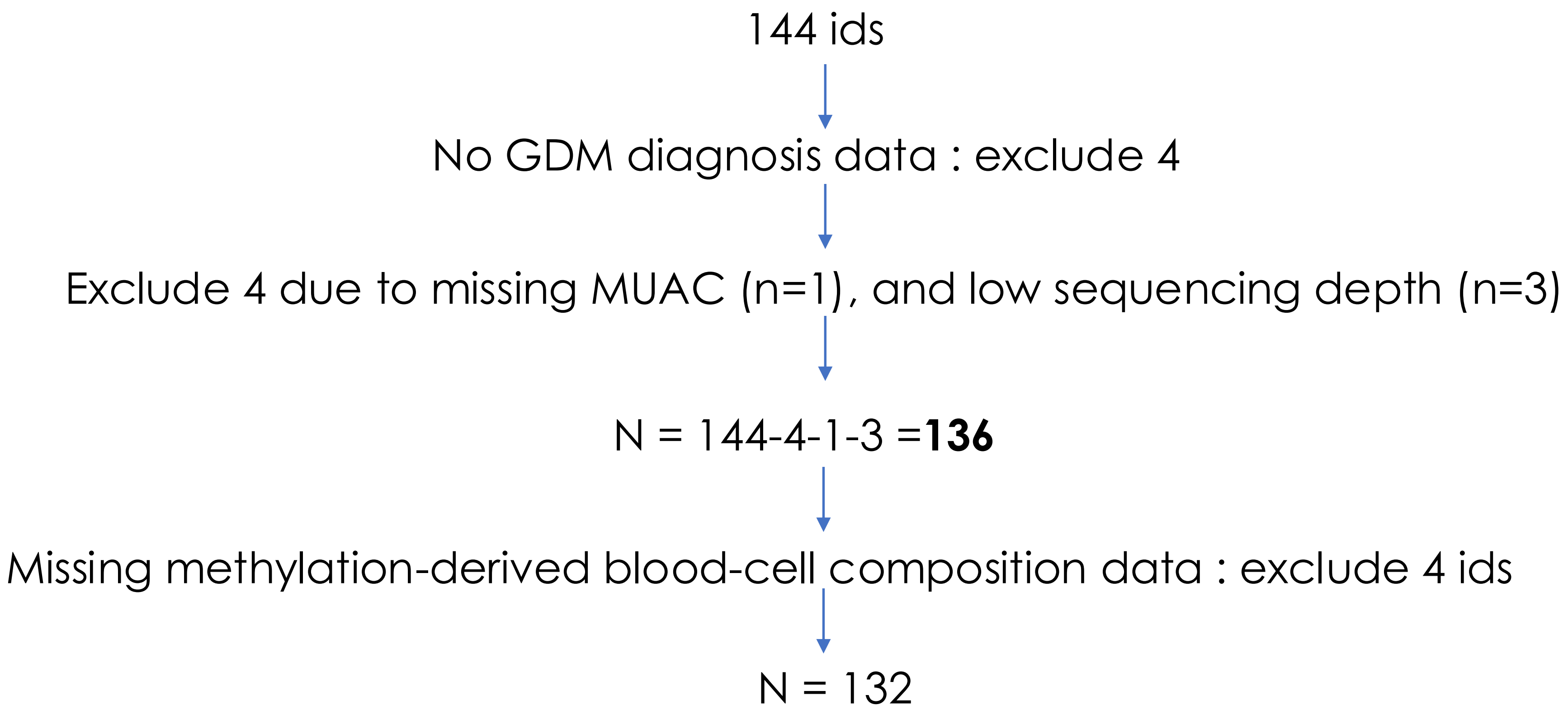

Supplementary Figure 1. STROBE chart

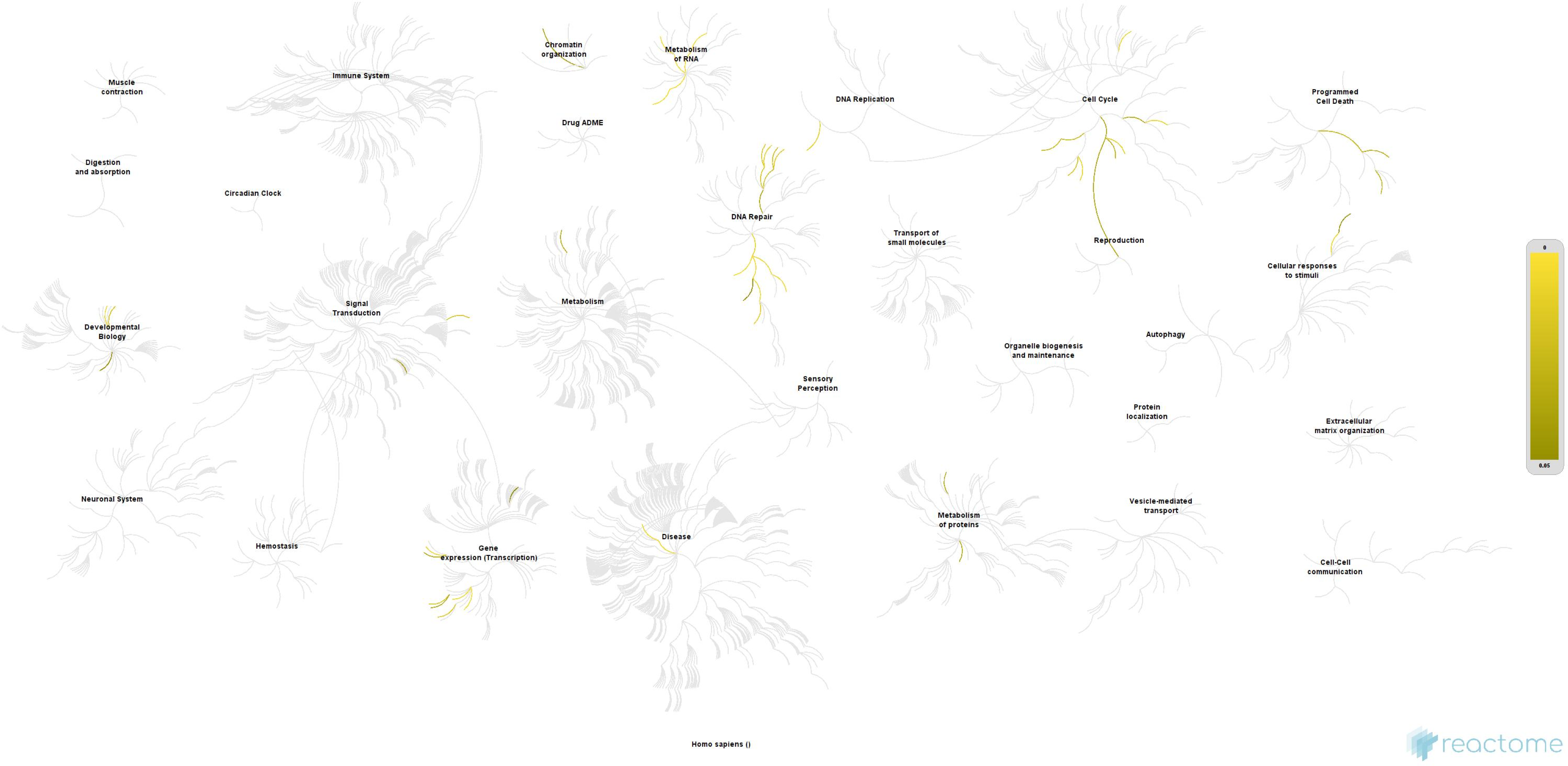
Muscle contraction
Immune System
Chromatin organization
Metabolism of RNA
DNA Replication
Cell Cycle
Programmed Cell Death
Drug ADME
Digestion and absorption
Circadian Clock
DNA Repair
Transport of small molecules
Reproduction
Cellular responses to stimuli
Developmental Biology
Signal Transduction
Metabolism
Autophagy
Organelle biogenesis and maintenance
Sensory Perception
Protein localization
Extracellular matrix organization
Neuronal System
Hemostasis
Gene expression (Transcription)
Disease
Metabolism of proteins
Vesicle-mediated transport
Cell-Cell communication
0
0.05
Homo sapiens ()
reactome
